# Photo Quiz: A cutaneous fungal infection with discordant biomarker results—a diagnostic challenge

**DOI:** 10.1128/jcm.02075-24

**Published:** 2025-06-11

**Authors:** Guillaume Dumont, Sofiane Chaib, Emmanuelle Bontemps, Marjorie Brottier, Agnes Meybeck, Marjorie Cornu, Dea Garcia-Hermoso, Pierre Patoz

**Affiliations:** 1 Laboratory of Microbiology, Gustave Dron Hospital, Tourcoing, France; 2 Infectious Diseases Unit, Gustave Dron Hospital, Tourcoing, France; 3 Intensive Care Unit, Gustave Dron Hospital, Tourcoing, France; 4 Laboratory of Parasitology-Mycology, University Hospital of Lille, Lille, France; 5 Institut Pasteur, French National Reference Center for Invasive Mycoses and Antifungals, Molecular Mycology Unit, Paris, France; Mayo Clinic Minnesota, Rochester, Minnesota, USA

## PHOTO QUIZ 

A 68-year-old woman native of Cameroon, who arrived in France 4 months before, was admitted to the Infectious Diseases Department of the Hospital of Tourcoing, France, for a suspicion of Mpox virus infection. She was recently diagnosed with an HIV infection, with a CD4 T-cell count of 10 per mm^3^, and presented with a chronic vesicular skin rash. The patient’s general condition progressively deteriorated, accompanied by asthenia, a dry cough, and worsening dyspnea.

**Fig 1:**
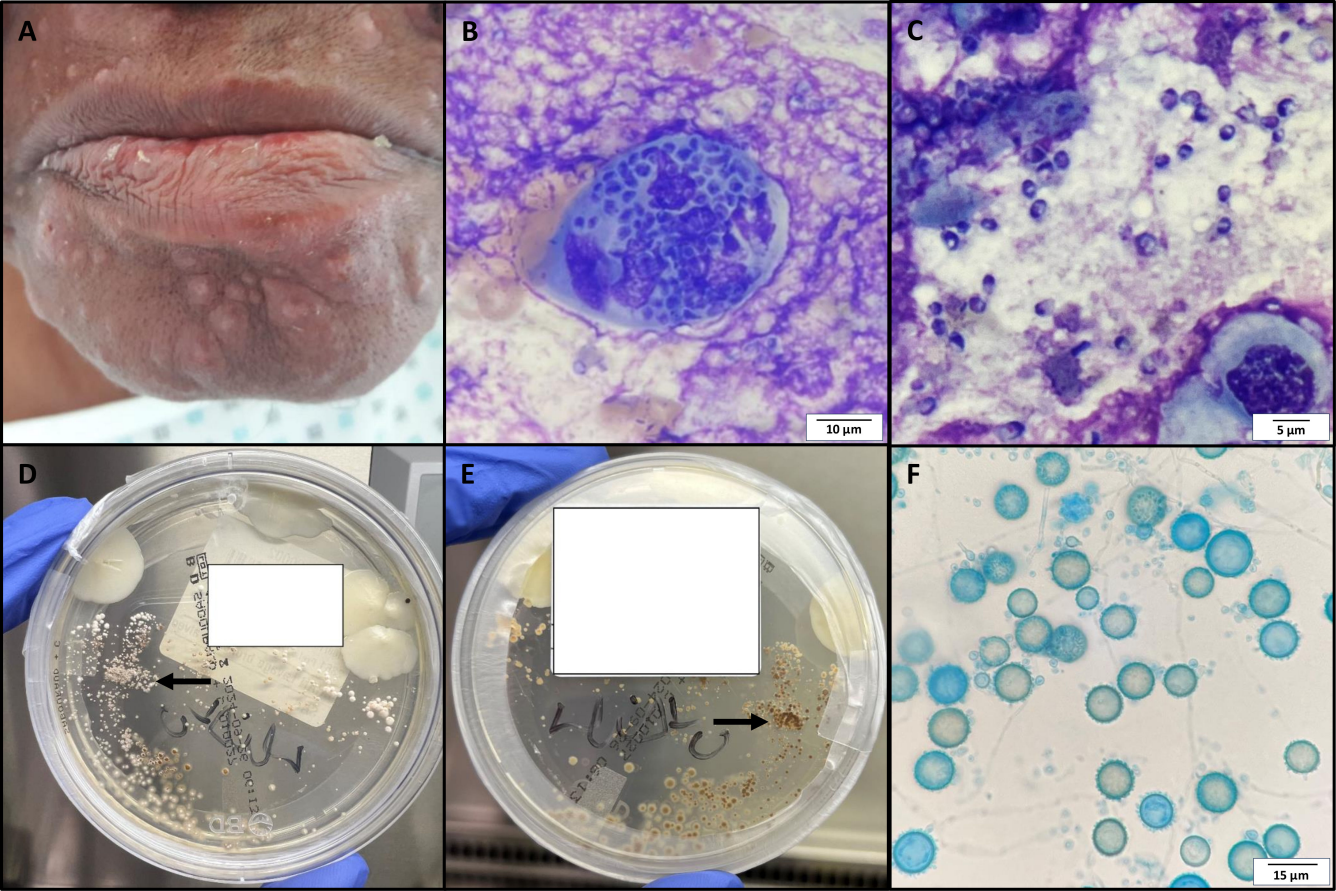
(**A**) Photograph of the perioral vesicular eruptions. (**B and C**) May-Grünwald Giemsa staining of the bronchial fraction of the bronchoalveolar lavage (magnification, ×1,000). (**B**) Visualization of intracellular yeasts measuring 1–3 µm. (**C**) Visualization of extracellular yeasts. (**D and E**) Sabouraud agar culture at 30°C after 12 days; as the plate is mixed with *Candida albicans* colonies, the arrow indicates the suspected filamentous fungus. (**D**) Front view of the agar plate. (**E**) Reverse view of the agar plate. (**F**) Cellophane tape preparation from culture growth stained with lactophenol aniline blue dye (magnification, ×1,000).

Upon examination, multiple non-pruritic vesicular lesions of varying ages were observed in the perioral and chin regions (Fig. 1A), with intraoral lesions noted, which may be the cause of the dysphagia that the patient was also presenting. The patient reported the onset of these vesicular eruptions 1 month before, with progressive extension. However, no clinical findings suggested an infection with the Mpox virus.

Laboratory tests revealed bicytopenia with anemia (hemoglobin 9.1 g/dL) and leukopenia (2.96 G/L). The quantification of the HIV RNA viral load (real-time PCR, HIV Alinity m reagent, Abbott) showed a viral load of over 4 million copies per milliliter.

Herpes simplex virus type 1 and 2 (HSV1-2) polymerase chain reaction (PCR) assay on skin swab (BioGX viral meningitis HSV/VZV OSR for BD MAX) was negative. In addition, the result of the *Histoplasma* serology test (double immunodiffusion, Meridian Bioscience) was negative. The detection of (1, 3)-beta-D-glucan (Fungitell assay, Associates of CAPE COD) in serum was also negative.

A bronchoalveolar lavage (BAL) was performed due to the dry cough and respiratory discomfort presented by the patient. In addition, the chest CT scan revealed bilateral diffuse interstitial pneumonia (DIP). Quantitative PCR for *Pneumocystis jirovecii* (BioGX *Pneumocystis jirovecii*, msg gene, OSR for BD MAX) and May-Grünwald Giemsa (MGG) staining of the alveolar fraction resulted in a diagnosis of *Pneumocystis jirovecii* pneumonia.

Fortuitously, the MGG straining of the bronchial fraction revealed the presence of numerous small (1–3 µm) thick-walled intra- and extracellular yeasts, which led to the suspicion of a concomitant other fungal infection (Fig. 1B and C).

The fungal cultures of the bronchial fraction were incubated at 30°C and yielded positive results 12 days later. The slow-growing colonies were white-beige, downy, and with a brown reverse (Fig. 1D and E). A tape mount performed on colonies showed septate hyaline filaments and numerous spherical, tuberculate, single-celled macroconidia (Fig. 1F).

What is your diagnosis?

## ANSWER TO PHOTO QUIZ

The patient was diagnosed with mucocutaneous histoplasmosis in the context of advanced HIV disease.

Figure 1B in the photo quiz illustrates a macrophage containing *Histoplasma capsulatum* yeasts. The characteristic oval-shaped spores, ranging from 1 to 3 µm, with a colorless vacuole that reduces the fungal cytoplasm to a small crescent, allowed for a presumptive diagnosis of histoplasmosis. Both macroscopic and microscopic features in culture strongly suggested *H. capsulatum*, a dimorphic fungal pathogen, with the microscopic appearance of the yeasts specifically pointing toward the *capsulatum* variety.

Proteomic analysis using MALDI-TOF mass spectrometry (Vitek MS PRIME, bioMerieux), with IVD database and MSI-2 application, confirmed the identification of *H. capsulatum*. The identification performed at the French National Reference Center for Invasive Mycoses identified the strain as *H. capsulatum*. A subsequent skin biopsy revealed *Histoplasma capsulatum* in both culture and histopathological examination, confirming mucocutaneous histoplasmosis. It is likely that the positive result in the bronchial fraction was due to oral contamination from the mucocutaneous lesions. Galactomannan antigen testing was conducted retrospectively on the initial serum sample, and a positive result was obtained with a galactomannan antigen concentration of 0.95 (using the Platelia Aspergillus Ag ELISA, Bio-Rad; threshold ≥0.5). The pulmonary involvement was attributed to *Pneumocystis jirovecii* rather than *H. capsulatum*, as both the MGG staining and the culture on the alveolar fraction were negative for *Histoplasma*. Regarding the extension work-up, brain MRI and cerebrospinal fluid analysis did not show any lesions or involvement by *H. capsulatum*. However, the abdominal-pelvic CT scan revealed mesenteric lymphadenopathy with a necrotic center, which underwent exploratory laparoscopy. The resection of the mesenteric lymphadenopathy, biopsied, revealed lymph node involvement by *H. capsulatum*, confirmed by both culture and histopathological analysis. The presence of lesions in non-contiguous body sites, along with systemic symptoms, is consistent with disseminated histoplasmosis, a condition in *which H. capsulatum* spreads beyond the respiratory system to various organs and tissues. This suggestion is supported by the positivity of galactomannan.

The treatment regimen included liposomal amphotericin B once a day (5 mg/kg/day) for 6 weeks as a slow intravenous injection, followed by long-term itraconazole 600 mg/day, or 3 capsules of 100 mg twice a day (trough concentration 0,5–1 mg/L).

*Histoplasma capsulatum* var. *capsulatum* is a dimorphic fungus responsible for histoplasmosis, primarily endemic to the Ohio and Mississippi River valleys in the United States, as well as some regions in Latin America, Africa, and Asia. The mode of transmission is primarily via inhalation of microconidia from contaminated environments, especially soil enriched with bird or bat droppings. The infection typically presents in various forms, including acute pulmonary histoplasmosis, chronic pulmonary histoplasmosis, and disseminated histoplasmosis, particularly affecting immunocompromised individuals (1). Histoplasmosis, particularly due to *Histoplasma capsulatum* var. *capsulatum*, presents a significant global health challenge, with recent estimates suggesting an annual incidence of approximately 100,000 cases of disseminated histoplasmosis worldwide (2).

The diagnosis of histoplasmosis in HIV-infected individuals residing in non-endemic regions presents considerable challenges. The gold standard for diagnosing invasive histoplasmosis remains fungal culture, although the diagnostic process can take several weeks. When available, serum or urine antigen testing for *Histoplasma* offers a valuable tool for early diagnosis (3). The 2024 French guidelines recommend performing *Histoplasma* serum or urinary antigen tests in HIV-infected patients from endemic areas, particularly those with initial CD4 T-cell counts <200 /µL (4). In the absence of these tests, alternative fungal biomarkers should be considered. For instance, galactomannan antigen testing, which is known to cross-react in case of *H. capsulatum* infection, should be employed in suspected cases (5). In our case, the initial positivity of this marker could have supported the diagnosis of histoplasmosis. In addition, (1, 3)-beta-D-glucan, a pan-fungal marker, can also help diagnose invasive histoplasmosis, with a sensitivity that seems to be close to 100% for disseminated forms (6). Furthermore, a negative *Histoplasma* serology should not rule out the diagnosis, as false-negative results are frequent due to the reduced sensitivity of antibody detection in individuals with advanced HIV (around 80% in immunocompetent individuals and 50% in advanced HIV disease patients) (3, 7).

Finally, in cases of clinical suspicion of histoplasmosis, it is essential to implement strict hygiene precautions in the laboratory. Indeed, samples suspected to contain *H. capsulatum* can be handled under biosafety level 2 (BSL-2) conditions, while growing cultures must be handled in a BSL-3 laboratory. The results of fungal markers should not undermine the strict application of these measures. Moreover, it is crucial to provide clinical information along with the samples to ensure proper management of the specimens.
